# Increasing access to mental health supports for 12–17-year-old Indigenous youth with the JoyPop mobile mental health app: study protocol for a randomized controlled trial

**DOI:** 10.1186/s13063-024-08076-y

**Published:** 2024-04-04

**Authors:** Aislin R. Mushquash, Teagan Neufeld, Ishaq Malik, Elaine Toombs, Janine V. Olthuis, Fred Schmidt, Crystal Dunning, Kristine Stasiuk, Tina Bobinski, Arto Ohinmaa, Amanda Newton, Sherry H. Stewart

**Affiliations:** 1 Department of Psychology, Lakehead University, Thunder Bay, Canada; 2 Dilico Anishinabek Family Care, Fort William First Nation, Canada; 3 Department of Psychology, University of New Brunswick, Fredericton, Canada; 4 Children’s Centre Thunder Bay, Thunder Bay, Canada; 5 Thunder Bay Counselling Centre, Thunder Bay, Canada; 6 Ontario Native Women’s Association, Thunder Bay, Canada; 7 School of Public Health, University of Alberta, Edmonton, Canada; 8 Department of Pediatrics, University of Alberta, Edmonton, Canada; 9 Departments of Psychiatry and Psychology and Neuroscience, Dalhousie University, Halifax, Canada

**Keywords:** Emotion regulation, Mental health, Youth, Indigenous, First Nations, Rural, eHealth, mHealth

## Abstract

**Background:**

Indigenous youth in Northwestern Ontario who need mental health supports experience longer waits than non-Indigenous youth within the region and when compared to youth in urban areas. Limited access and extended waits can exacerbate symptoms, prolong distress, and increase risk for adverse outcomes. Innovative approaches are urgently needed to provide support for Indigenous youth in Northwestern Ontario. Using a randomized controlled trial design, the primary objective of this study is to determine the effectiveness of the JoyPop app compared to usual practice (UP; monitoring) in improving emotion regulation among Indigenous youth (12–17 years) who are awaiting mental health services. The secondary objectives are to (1) assess change in mental health difficulties and treatment readiness between youth in each condition to better understand the app’s broader impact as a waitlist tool and (2) conduct an economic analysis to determine whether receiving the app while waiting for mental health services reduces other health service use and associated costs.

**Methods:**

A pragmatic, parallel arm randomized controlled superiority trial will be used. Participants will be randomly allocated in a 1:1 ratio to the control (UP) or intervention (UP + JoyPop) condition. Stratified block randomization will be used to randomly assign participants to each condition. All participants will be monitored through existing waitlist practices, which involve regular phone calls to check in and assess functioning. Participants in the intervention condition will receive access to the JoyPop app for 4 weeks and will be asked to use it at least twice daily. All participants will be asked to complete outcome measures at baseline, after 2 weeks, and after 4 weeks.

**Discussion:**

This trial will evaluate the effectiveness of the JoyPop app as a tool to support Indigenous youth waiting for mental health services. Should findings show that using the JoyPop app is beneficial, there may be support from partners and other organizations to integrate it into usual care pathways.

**Trial registration:**

https://clinicaltrials.gov/study/NCT05898516 [registered on June 1, 2023].

## Administrative information

Note: the numbers in curly brackets in this protocol refer to SPIRIT checklist item numbers. The order of the items has been modified to group similar items (see http://www.equator-network.org/reporting-guidelines/spirit-2013-statement-defining-standard-protocol-items-for-clinical-trials/).

**Table:** 

Title {1}	Increasing access to mental health supports for 12–17 year-old Indigenous youth with the JoyPop mobile mental health app: study protocol for a randomized controlled trial
Trial registration {2a and 2b}.	ClinicalTrials.gov NCT05898516. Registered on June 1, 2023.
Protocol version {3}	December 13, 2023; version 1
Funding {4}	Canadian Institutes of Health Research/Sick Kids Foundation—New Investigator Research Grant (ARM) In-kind support provided by Dilico Anishinabek Family Care Tier 1 Canada Research Chair in Addictions and Mental Health (SHS)
Author details {5a}	ARM: Department of Psychology, Lakehead University; Dilico Anishinabek Family Care TN: Department of Psychology, Lakehead University IM: Department of Psychology, Lakehead University ET: Department of Psychology, Lakehead University; Dilico Anishinabek Family Care JO: Department of Psychology, University of New Brunswick FS: Department of Psychology, Lakehead University; Children’s Centre Thunder Bay CD: Thunder Bay Counselling Centre KS: Dilico Anishinabek Family Care TB: Ontario Native Women’s Association AO: School of Public Health, University of Alberta AN: Department of Pediatrics, University of Alberta SHS: Departments of Psychiatry and Psychology & Neuroscience, Dalhousie University ARM is the principal investigator and conceived the study and led the protocol development. ET, JO, FS, AN, and SHS provided substantial contributions to inform the study design. ARM, ET, JO, AO, FS, AN, and SHS collaborated to seek funding to support the study. ET, FS, CD, KS, and TB provided feedback about local implementation, recruitment, and retention strategies. ARM supervised all aspects of the trial. TN led the trial coordination and data collection with support from IM, ET, and ARM. ARM drafted the manuscript. All authors reviewed and approved the final manuscript.
Name and contact information for the trial sponsor {5b}	Aislin R. Mushquash Department of Psychology, Lakehead University 955 Oliver Road, Thunder Bay, Ontario, P7B5E1 Email: aislin.mushquash@lakeheadu.ca
Role of sponsor {5c}	Funders have no role in the study design, data collection, analysis, or management, or reports/publication.

## Introduction

### Background and rationale {6a}

Northwestern Ontario, Canada, is situated within the Nishnawbe Aski Nation and is home to 49 Indigenous communities. Indigenous youth within this region, and generally, are exposed to circumstances that increase their risk for mental health difficulties including histories of family residential school attendance [1], early childhood adversity [2], and economic and housing instability [3]. As a result, Indigenous youth experience more mental health difficulties than non-Indigenous youth [4]. Indigenous youth within Northwestern Ontario experience longer waits for mental health services when compared to non-Indigenous youth within the region and in other areas of the country [5–7]. These issues are concerning as extended time on waitlists can exacerbate symptoms, prolong distress, and increase risk for more adverse outcomes [8–11]. Delayed access to mental health services also negatively impacts treatment engagement [9, 12], with non-attendance, decreased motivation, and reduced satisfaction with services frequently reported following prolonged wait times [9, 11].

Innovative approaches are urgently needed to provide support for Indigenous youth. Making use of emerging technologies (e.g., mobile health apps) to provide mental health supports is discussed as holding particular promise for youth, for those living in rural and remote areas, and for Indigenous people [13, 14]. With these “just-in-time” interventions [15], support can be accessed in-the-moment, when it is needed most [16]. However, despite their potential, significant gaps exist between the growing number of apps available in the public domain and empirical demonstration of the beneficial impacts of apps for users [17–19]. For example, a recent review found that only 3.4% of available mobile mental health apps had research to support the claims made about their effectiveness [20]. More studies on the effectiveness of youth-oriented mobile mental health apps among Indigenous youth are especially needed before providers adopt or integrate them into usual care pathways [21].

**Fig. 1:**
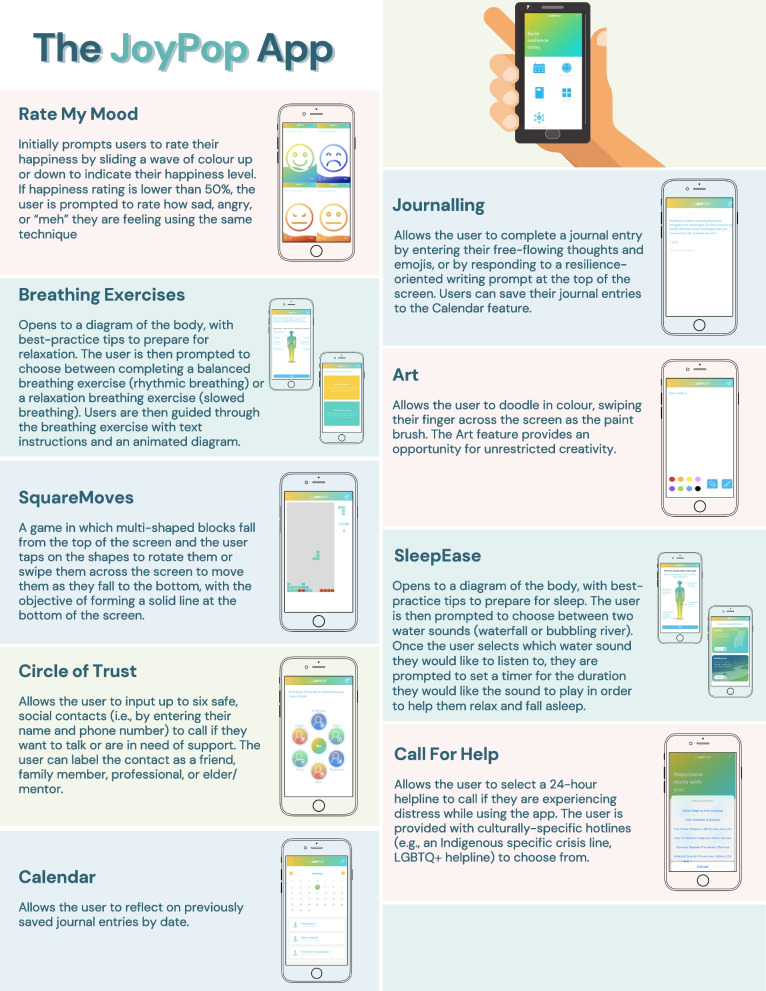
The JoyPop app

This research is developed in partnership with an Indigenous-led community organization that is guided by Anishinabek culture and which supports Indigenous children, families, and communities within Northwestern Ontario. Our team is evaluating the impact of a mobile mental health app (JoyPop; see Fig. 1) as a tool for Indigenous youth waiting for mental health services. While using an app is unlikely to alleviate all mental health difficulties among youth seeking services, it could serve as a skill-building tool that improves emotion regulation, reduces mental health symptoms, and increases motivation prior to treatment, thus contributing to a better treatment response [11, 22, 23].

The JoyPop app was developed using patient-oriented research methods involving active collaboration between youth, service providers, and clinician-scientists [24]. The app incorporates evidence-based features that support improved emotion regulation [25]. JoyPop’s focus on emotion regulation reflects current recommendations for mental health interventions to focus on transdiagnostic factors rather than addressing individual disorders in isolation [26] and has relevance to Indigenous youth in Northwestern Ontario who typically present with difficulties related to emotion regulation [27].

The JoyPop app has a growing evidence base that shows important benefits or potential for youth [25]. MacIsaac et al. examined the impact of the JoyPop app among older youth in university [25] and found that with each day of app use, there was a corresponding positive change in emotion regulation and mental health [25]. Improvements were especially evident for those with more childhood adversity (e.g., abuse, neglect). Mushquash et al. examined feedback from university students who used the app for up to 4 weeks [28] and found that most students described experiencing positive outcomes (e.g., improved emotional expression) after using the app. Kim et al. gathered input from Indigenous community members and stakeholders and found that most saw potential for the app to foster resilience among youth [29]. Finally, Malik et al. evaluated the acceptance of the app among youth (12–18 years) receiving mental health services and among service providers, both in Northwestern Ontario [30]. Youth and service providers perceived the app as useful because it facilitated accessibility to coping skills. All participants expressed positive attitudes and feelings towards the app because of the benefits it provided youth or the advantages it could provide to local mental health services.

### Objectives {7}

The primary objective of this study is to determine the effectiveness of the JoyPop app compared to usual practice (UP; monitoring) in improving emotion regulation among Indigenous youth (12–17 years) awaiting mental health services. Based on our past research [25, 28], we hypothesize that youth receiving the app will show significantly greater improvement in emotion regulation compared to youth receiving only UP.

The secondary objectives are to (1) compare change in mental health difficulties and treatment readiness between youth in each condition to better understand the app’s broader impact as a waitlist tool and (2) conduct an economic analysis to determine whether receiving the app while waiting for mental health services reduces other health service use and associated costs.

### Trial design {8}

A pragmatic, parallel arm randomized controlled superiority trial will be used. Participants will be randomly allocated in a 1:1 ratio to the control (usual practice; UP) or intervention (UP + JoyPop) condition. Stratified block randomization will be used to randomly assign participants to each condition. All participants will be monitored through existing waitlist management practices, which involve regular phone calls to check in and assess functioning. Participants in the intervention condition will receive access to the JoyPop app for 4 weeks and be asked to use it at least twice daily. All participants will be asked to complete primary (emotion regulation) and secondary (mental health symptoms, treatment readiness, service utilization) outcome measures at pre (baseline), mid (after 2 weeks), and post (after 4 weeks) timepoints.

The Standard Protocol Items: Recommendations for Intervention Trials (SPIRIT) guidelines are being followed. The trial was registered with ClinicalTrials.gov (NCT05898516). All procedures have been reviewed and approved by the Research Ethics Board at the Thunder Bay Regional Health Sciences Centre (acting as the Board of Record for Lakehead University clinical research projects) and the Research Advisory Committee at our partner organization, Dilico Anishinabek Family Care (“Dilico”).

## Methods: participants, interventions, and outcomes

### Study setting {9}

Youth on the waitlist for mental health services at Dilico, located in Northwestern Ontario, Canada, will be recruited. Data collection will occur at Dilico. Dilico provides a range of responsive individual, family, and community programs and services for the complete life journey of Anishinabek people. Dilico is a self-governed organization that is recognized as a leader in research and delivery of child welfare, mental health and addictions, and health services. The community-based services delivered by Dilico are designed to enhance the well-being of Anishinabek children, families, and communities in a culturally safe manner [31].

### Eligibility criteria {10}

Youth are eligible if they are on the waitlist for mental health services with Dilico and are between 12 and 17 years old. If participants cannot attend a session at Dilico (e.g., due to weather), accommodations will be offered (e.g., to hold the session virtually).

### Who will take informed consent? {26a}

Interested participants will attend an orientation session at Dilico. During the session, a research assistant will present information about the study and provide a copy of an information letter that contains all relevant details. Each potential participant will have time to review the information letter and ask questions. Participants will then be provided with the informed consent form to review and complete. Potential participants are deemed capable of providing consent independently. For youth under 16 years, the research assistant will encourage youth of the desirability of informing their parent(s)/caregiver(s) about their involvement in the research; however, parental/caregiver involvement/consent is not required. This approach is consistent with the Canadian Tri-Council Policy Statement: Ethical Conduct for Research Involving Humans [32], with the practices in place at Dilico Anishinabek Family Care, and with youth 12 years old and up being able to consent to counseling services independently via the provincial Child, Youth and Family Services Act [33].

### Additional consent provisions for collection and use of participant data and biological specimens {26b}

No additional consent provisions are in place. No biological samples will be collected.

## Interventions

### Explanation for the choice of comparators {6b}

The control condition (UP) was chosen to account for the potential effects of time on emotion regulation. It will be used to test whether receiving the JoyPop app is associated with improved outcomes compared to UP.

### Intervention description {11a}

The control condition (UP) involves participants being monitored through existing waitlist management practices in place at Dilico (i.e., regular phone calls to check in and assess functioning). The intervention condition (UP + JoyPop) involves participants being monitored through existing waitlist management practices in place at Dilico plus receiving access to the JoyPop app. The app is currently only available for iOS devices (iPad, iPhone, iPod). If participants do not have access to their own iOS device, they will be provided with a refurbished iPhone for the duration of the study. The refurbished iPhones contain only the JoyPop app (no other features available/enabled). Participants in the intervention condition will be asked to use the app at least twice daily for 4 weeks but will otherwise not be provided with requirements related to app usage. The app incorporates evidence-based features that support improved emotion regulation [25] including Rate My Mood, Journaling, Breathing Exercises, Art, SquareMoves, SleepEase Circle of Trust, and Call for Help (see Fig. 1 for description of features).

### Criteria for discontinuing or modifying allocated interventions {11b}

Any participant can request to discontinue the intervention by contacting the research team. At baseline, participants will not only complete outcome measures but will also complete a demographics measure and psychiatric symptoms screening measure. These measures will be used to describe the sample and better understand who is accessing the research study. If a participant endorses recent suicidal thoughts on the symptoms screening measure, the research assistant will notify the clinical services case manager from Dilico who will identify a counselor to conduct a suicide risk assessment with the participant and provide any necessary immediate supports. The participant and counselor will determine whether continuing with the study is recommended. There are no pre-determined criteria that would require a participant to discontinue the allocated intervention.

### Strategies to improve adherence to interventions {11c}

Our pilot research showed that having in-person contact with a consistent research assistant was important for participant engagement [34]. Therefore, all participants in this study will meet with a research assistant when they attend an orientation session at the beginning of the study. Participants in the intervention condition will receive text and email reminders to use the app each day. Text and email reminders will be automated to arrive each day at 8 am and 8 pm. Participants in both conditions will also receive text and email reminders to attend sessions to complete pre, mid, and post outcome measures. Participants will receive cash compensation for each session that they attend ($20 baseline; $25 mid; $30 post) and an additional cash incentive ($25) if they attend all three sessions. All participants will be encouraged to reach out to the research team with any questions or concerns throughout the study.

### Relevant concomitant care permitted or prohibited during the trial {11d}

Participants are permitted to receive concomitant care as needed. No concomitant care and interventions are prohibited during the trial. Concomitant care will be assessed through the outcome measures completed by participants.

### Provisions for post-trial care {30}

All participants remain on the waitlist for services with Dilico; thus, most will likely receive care post-intervention (unless they choose not to once services are offered). There are no expected trial-related harms.

### Outcomes {12}

#### Primary outcomes

The primary outcome is change in emotion regulation skills from pre (baseline) to mid (after 2 weeks) and post (after 4 weeks). Emotion regulation will be assessed with the Difficulties in Emotion Regulation Scale—Short Form (DERS-SF) [35, 36]. The DERS-SF is 18 items and asks respondent how often specific statements applied to them over the previous 2 weeks on a 5-point scale ranging from 1 (*almost never*) to 5 (*almost always*). We will examine change in overall emotion regulation via the total DERS-SF score and change in specific domains via the DERS-SF subscales. Total scores range from 18 to 90 with higher scores indicating greater difficulties in emotion regulation. Subscales are as follows: strategies (e.g., “When I was upset, I believed there was nothing I could have done to make myself feel better”), non-acceptance (e.g., “When I was upset, I became irritated with myself for feeling that way”), impulse (e.g., “When I was upset, I became out of control”), goals (e.g., “When I was upset, I had difficulty getting work done”), awareness (e.g., “I paid attention to how I feel”), and clarity (e.g., “I had no idea how I was feeling”). Total subscale scores range from 3 to 15 with higher scores indicating greater difficulty.

#### Secondary outcomes

Secondary outcomes include change in mental health difficulties, treatment readiness, and service utilization from pre (baseline) to mid (after 2 weeks) and post (after 4 weeks). Mental health difficulties will be assessed with the Depression, Anxiety, and Stress Scale 21 (DASS-21) [37] and the Strengths and Difficulties Questionnaire (SDQ) [38, 39]. Treatment readiness will be assessed with the treatment readiness subscale of the Motivation for Youth’s Treatment Scale (MYTS) [40]. Service utilization will be assessed with a researcher-generated scale.

The DASS-21 is 21 items and asks respondents how much specific statements applied to them over the previous week on a 4-point scale ranging from 0 (*never*) to 3 (*almost always*). We will examine change in overall psychological distress via the total DASS-21 score and change in specific domains via the three 7-item DASS-21 subscales. Total scores range from 0 to 63 with higher scores indicating greater psychological distress. Subscales are as follows: depressive symptoms (e.g., “I couldn’t seem to experience any positive feeling at all”), anxious symptoms (e.g., “I was aware of dryness of my mouth”), and stress (e.g., “I found it hard to wind down”). Subscale scores range from 0 to 21 with higher scores indicating greater symptoms/stress.

The SDQ is 25 items and asks respondents how true an item is for them over the last 6 months on a 3-point scale ranging from 0 (*not true*) to 2 (*certainly true*). We will examine change in overall difficulties via the total SDQ score (with exception of prosocial items) and change in specific strengths and difficulties via the SDQ subscales. Total scores range from 0 to 40 with higher scores indicating more difficulties. Subscales are as follows: emotional problems (e.g., “I worry a lot”), conduct problems (e.g., “I get very angry and often lose my temper”), hyperactivity (e.g., “I am constantly fidgeting or squirming”), peer problems (e.g., “I would rather be alone than with people of my age”), and prosocial behavior (e.g., “I try to be nice to other people. I care about their feelings”). Subscale scores range from 0 to 10 with higher scores indicating more difficulties (except the prosocial subscale where lower scores indicate more difficulties).

The treatment readiness subscale of MYTS is 4 items (e.g., “I want help finding solutions for my current problems”) and asks respondents how strongly they agree or disagree with each statement on a 5-point scale ranging from 1 (*strongly disagree*) to 5 (*strongly agree*). The subscale score ranges from 4 to 20 with higher scores indicating greater readiness.

The service utilization scale is 5 items and asks respondents about the frequency of healthcare services utilization (i.e., number of visits for/with: walk in clinic, family doctor or nurse practitioner, emergency department, mental health counselor, mental health hotline/phone support) over the prior 2 weeks. Items will be analyzed individually and as a total score across service types.

#### Exploratory measures

Exploratory outcomes include an evaluation of app quality at post (after 4 weeks) among the intervention group. App quality will be assessed with the User Version of the Mobile Application Rating Scale (uMARS) [41]. The uMARS is 27 items and asks respondents to rate the quality of the app across each item on a 5-point scale ranging from 1 (*inadequate*) to 5 (*excellent*). We will evaluate app quality overall via the total uMARS score and app quality within specific domains via the uMARS subscales. Total scores range from 1 to 5 with higher scores indicating greater quality. Subscales are as follows: engagement (e.g., “Is the app fun/entertaining to use? Does it have components that make it more fun than other similar apps?”), functionality (e.g., “How accurately/fast do the app features (functions) and components (buttons/menus) work?”), esthetics (e.g., “How high is the quality/resolution of graphics used for buttons, icons, menus and content?”), information (e.g., “Is app content correct, well written, and relevant to the goal/topic of the app?”), subjective (e.g., “Would you recommend this app to people who might benefit from it?”), and perceived impact (e.g., “This app has increased my awareness of the importance of addressing the health behavior”). Subscale scores range from 1 to 5 with higher scores indicating more difficulties.

#### Descriptive measures

Descriptive information will be obtained through a demographic questionnaire at pre (baseline). Items will assess age, ethnicity, family composition, highest level of education, gender, sex at birth, sexual orientation, and living situation. Symptom presentation will be assessed with the DSM-5 Self-Rated Level 1 Cross-Cutting Symptoms Measures [42] at pre (baseline). The original measure is 25 items; however, we removed one item related to previous suicide attempts as per our community partner’s request. Respondents are asked to rate how much (or how often) they have been bothered by the specific symptoms during the past 2 weeks. Nineteen of the items are rated on a 5-point scale (*0* = *none or not at all; 1* = *slight or rare, less than a day or two; 2* = *mild or several days; 3* = *moderate or more than half the days; and 4* = *severe or nearly every day*). The suicidal ideation and substance abuse items are rated using a “Yes or No” scale. Scores will be summarized across the 12 psychiatric domains (i.e., depression, anger, irritability, mania, anxiety, somatic symptoms, inattention, suicidal ideation, psychosis, sleep disturbance, repetitive thoughts and behaviors, and substance use) and used to characterize the sample.

### Participant timeline {13}



### Sample size {14}

Our past research suggests a medium effect for the primary outcome [43]. We estimated conservatively in calculating sample size, using *f* = 0.2 (small to medium effect), alpha = 0.05, and power = 0.95 for a 2 (between subjects; treatment condition) by 3 (within subjects; time) mixed design, which approximates the statistical power needed for the planned linear mixed model under the assumption of compound symmetry (i.e., homogeneity of variance and covariance) [44]. Results suggested a sample size of 66 would achieve necessary power. Given our pilot data [34], we estimate 60% retention throughout the study (40% attrition), meaning we will need an initial sample size of 110.

### Recruitment {15}

All eligible youth will be informed about the JoyPop app and the study by waitlist case managers through various modalities including mailed and e-mailed letters/flyers and phone calls. Youth will be informed about the study once they are added to the waitlist for mental health service at Dilico and periodically while they wait for services (i.e., when waitlist case managers contact clients to check in).

## Assignment of interventions: allocation

### Sequence generation {16a}

Participants will be randomly allocated in a 1:1 ratio to the control (UP) or intervention (UP + JoyPop) condition. Stratified block randomization will be used to randomly assign participants to each condition. Randomization will be conducted by a researcher not affiliated with the trial using a computer-generated sequencing tool [45].

### Concealment mechanism {16b}

Implementation of the allocation sequence will be managed by a researcher not affiliated with the trial. Once the sequence is generated by the sequencing tool, the researcher will place individual allocations into numbered, opaque, sealed envelopes. Envelopes will be stored in a lock box that only the research assistant determining allocation will have access to.

### Implementation {16c}

After participants complete the measures at pre (baseline), the research assistant will obtain and open the next sequenced envelope to determine the allocation and inform the participant.

## Assignment of interventions: blinding

### Who will be blinded {17a}

Given the nature of this trial, it is not possible to blind participants to their allocation. As measures are self-report, blinding of outcome measures is also not possible. Protection against bias will occur by blinding investigators to condition (i.e., only research assistants directly managing participants will be unblinded). The statistician conducting the analyses will be blinded to conditions.

### Procedure for unblinding if needed {17b}

Not applicable as participants and research assistants will not be blinded to condition.

## Data collection and management

### Plans for assessment and collection of outcomes {18a}

Data will be collected via hard-copy, self-report measures for participants in both conditions. For those in the intervention condition, app usage data will also be obtained through the back-end app database. The research assistant that meets with participants at Dilico will explain how to complete the measures and will be available to answer any questions. Measures are described above in the “Outcomes {12}” section. Measures can be found within the sources cited above.

### Plans to promote participant retention and complete follow-up {18b}

To support retention, participants receive reminders via text/email to return to Dilico and complete measures at each timepoint. In addition, participants will receive cash compensation at each timepoint ($20 baseline; $25 mid; $30 post), as cash incentives enhance the completion of outcome measures in randomized trials [46, 47]. While we had some success with this approach in our pilot studies [44], we will provide a bonus $25 if all timepoints are completed as a further incentive [48]. The principal investigator and research assistants will also have weekly meetings to discuss the trial and monitor retention to determine if additional strategies to promote retention are required. If transportation is a barrier for participants, a taxi will be booked and paid for (through in-kind contribution from our partner organization).

### Data management {19}

A research assistant will transfer participant responses from the hard-copy measures into an online questionnaire hosted via Survey Monkey. The information systems and technical infrastructure for Survey Monkey are hosted within SOC 2 accredited data centers [49]. Physical security controls at the data centers include 24 × 7 monitoring, cameras, visitor logs, entry requirements, and dedicated cages for Survey Monkey hardware. Survey Monkey encrypts data in transit using secure TLS cryptographic protocols. App data will be stored on a password-protected server within Canada. App data is encrypted during transmission. Upon receipt, app data will be saved and stored on a password protected computer. In accordance with the Ownership, Control, Access, and Possession (OCAP™) [50] standards set by the First Nations Information Governance Centre, all electronic data obtained from Indigenous youth will be stored on a password protected computer at Dilico. All hard-copy data will be stored in a locked filing cabinet at Dilico. In accordance with Lakehead University’s policy, data will be retained for at least 5 years following the completion of the research.

### Confidentiality {27}

Confidentiality will be maintained throughout the study in accordance with the Canadian Tri-Council Policy Statement: Ethical Conduct for Research Involving Humans [32] and Thunder Bay Regional Health Sciences Centre and Lakehead University ethical guidelines. All participants will be provided an ID number at the beginning of their participation. All data (surveys and app data) will contain only this ID number. The log linking participant IDs to identifiers will be stored separately from study data and destroyed following data collection. One limitation to confidentiality is if participants endorse recent suicidal thoughts on the symptoms screening measure. If this occurs, the research assistant will notify the clinical services case manager from Dilico. Participants will be informed about confidentiality, the limits to confidentiality, and measures taken to protect their confidentiality, during the informed consent procedure.

### Plans for collection, laboratory evaluation, and storage of biological specimens for genetic or molecular analysis in this trial/future use {33}

Not applicable. Biological specimens will not be collected.

## Statistical methods

### Statistical methods for primary and secondary outcomes {20a}

An independent, contracted, statistician will lead the analyses. Linear mixed modeling will be used to test hypotheses related to emotion regulation, mental health symptoms, and treatment motivation. Polynomial contrasts will be used to test for linear and quadratic changes over time. Effect sizes will be calculated using the Cohen’s *d* formula adapted by Feingold [51]. In the economic analysis, mental health services utilization and their costs during the 4-week period among participants in each condition will be estimated. Then, the incremental costs/savings associated with receiving the app using a cost-consequence analysis will be estimated [52].

### Interim analyses {21b}

Not applicable. No interim analyses are planned.

### Methods for additional analyses (e.g., subgroup analyses) {20b}

No additional analyses are planned. Subgroup analyses may be explored based on the composition of the final sample and the direction of our community partners.

### Methods in analysis to handle protocol non-adherence and any statistical methods to handle missing data {20c}

All randomized participants will be included in analyses (i.e., intent-to-treat). Missing data will be handled with full information maximum likelihood estimation—a preferred method that results in relatively unbiased parameters and valid model fit [53, 54].

### Plans to give access to the full protocol, participant-level data, and statistical code {31c}

The full trial protocol will be available publicly (e.g., through clinicaltrial.gov and BMC Trials). In accordance with OCAP™ [50], data will be retained by Dilico Anishinabek Family Care. Data will not be shared with any third parties.

## Oversight and monitoring

### Composition of the coordinating center and trial steering committee {5d}

Dr. Aislin Mushquash, as the principal investigator, will be responsible for oversight and management of all research activities. Dr. Mushquash will supervise and meet with the research assistants weekly. Research assistants include undergraduate and graduate students. Dr. Mushquash will consult with co-investigators quarterly to plan for and discuss ongoing trial management. Co-investigators include Dr. Janine Olthuis, Dr. Elaine Toombs, Dr. Fred Schmidt, Dr. Arto Ohinmaa, Dr. Amanda Newton, and Dr. Sherry H. Stewart. Dr. Mushquash will also consult with the local community partner collaborators quarterly regarding implementation. The trial steering committee includes collaborators Crystal Dunning, Kristine Stasiuk, and Tina Bobinski, along with the principal investigator and co-investigators.

### Composition of the data monitoring committee, its role and reporting structure {21a}

Investigators will monitor the trial. Dr. Aislin Mushquash will meet with the research assistants weekly to discuss recruitment, retention, and data collection. Given minimal risks associated with the intervention, a formal data monitoring committee was not developed.

### Adverse event reporting and harms {22}

No anticipated harms are associated with participating in the trial. If a participant feels upset during or after the study, they will be encouraged to contact relevant support services. Dr. Aislin Mushquash will inquire about adverse events during weekly meetings with research assistants. Research assistants are also directed to inform Dr. Aislin Mushquash of any concerns or adverse events as soon as they occur. If an adverse event were to occur, Dr. Aislin Mushquash would submit the Research Ethics Local Serious Adverse Event Reporting Form to the Thunder Bay Regional Health Sciences Centre Research Ethics Board.

### Frequency and plans for auditing trial conduct {23}

Dr. Aislin Mushquash will review protocol adherence weekly with research assistants. We have no plans for independent auditing of trial conduct.

### Plans for communicating important protocol amendments to relevant parties (e.g., trial participants, ethical committees) {25}

Protocol amendments would be submitted to the Thunder Bay Regional Health Sciences Centre Research Ethics Board for review and approval. Investigators would be informed via email or through quarterly meetings. Trial registries would be updated as needed. If needed and relevant, participants would be updated via phone or email.

### Dissemination plans {31a}

In accordance with OCAP™ [50], all results will be reviewed by Dilico Anishinabek Family Care’s Research Advisory Committee. If the committee approves, results will be shared more broadly (e.g., through conference presentations, peer-reviewed publications, media interviews). Summaries will also be shared (e.g., via infographic, via presentation) with staff from our community partner site. Participants will have the option to opt into receiving a summary of the findings as well.

## Discussion

In Northwestern Ontario, Indigenous youth access mental health services less often and have longer waits than youth in other areas of the province. Limited access and extended waits can lead to negative outcomes and impact engagement. There is recognition that innovative approaches are needed to help address mental health needs for Indigenous youth. In partnership with Dilico Anishinabek Family Care, we are evaluating the impact of a mobile mental health app (JoyPop) as a tool for youth who are waiting for mental health services. Should findings show that using the JoyPop app benefits Indigenous youth who are waiting for services, and is cost-effective, there may be support to continue offering it more broadly.

## Trial status

The trial study protocol was approved by the Research Ethics Board at the Thunder Bay Regional Health Sciences Centre (acting as the Board of Record for Lakehead University clinical research projects; # 100157) on December 16, 2022 (study protocol version 4). The study was renewed/re-approved on December 18, 2023 (study protocol version 4).

Trial start date: May 26, 2023.

Estimated completion date: December 2024.

## Data Availability

In accordance with the OCAP™ [50], data obtained from Indigenous youth will be owned, controlled, and managed through the partner organization Dilico Anishinabek Family Care.

## Abbreviations

UP: Usual practice
SPIRIT: Standard Protocol Items: Recommendations for Intervention Trials
Dilico: Dilico Anisinabek Family Care
iOS device: IPad, iPod, iPhone
DERS-SF: Difficulties in Emotion Regulation Scale—Short Form
DASS-21: Depression, Anxiety and Stress Scale 21
SDQ: Strengths and Difficulties Questionnaire
MYTS: Motivation for Youth’s Treatment Scale
uMARS: User Version of the Mobile Application Rating Scale
OCAP™: Ownership, Control, Access and Possession Standards™
